# Differential recognition of influenza A virus H1N1 neuraminidase by DNA vaccine-induced antibodies in pigs and ferrets

**DOI:** 10.3389/fimmu.2023.1200718

**Published:** 2023-05-29

**Authors:** Jeanette Linnea Tingstedt, Christine Stephen, Christian Risinger, Ola Blixt, Vithiagaran Gunalan, Isik Somuncu Johansen, Anders Fomsgaard, Charlotta Polacek, Ria Lassaunière

**Affiliations:** ^1^ Virus Research & Development Laboratory, Department of Virus & Microbiological Special Diagnostics Statens Serum Institut, Copenhagen, Denmark; ^2^ Research Unit of Infectious Diseases, Clinical Institute, University of Southern Denmark, Odense, Denmark; ^3^ Department of Chemistry and Biochemistry, University of California, San Diego, La Jolla, CA, United States; ^4^ Department of Chemistry, University of Copenhagen, Copenhagen, Denmark; ^5^ Department of Biotechnology and Biomedicine, Technical University of Denmark, Lyngby, Denmark

**Keywords:** influenza, neuraminidase, vaccines, DNA vaccine, antibodies, epitope mapping, neuraminidase inhibition, ELLA

## Abstract

Neuraminidase (NA) accounts for approximately 10-20% of the total glycoproteins on the surface of influenza viruses. It cleaves sialic acids on glycoproteins, which facilitates virus entry into the airways by cleaving heavily glycosylated mucins in mucus and the release of progeny virus from the surface of infected cells. These functions make NA an attractive vaccine target. To inform rational vaccine design, we define the functionality of influenza DNA vaccine-induced NA-specific antibodies relative to antigenic sites in pigs and ferrets challenged with a vaccine-homologous A/California/7/2009(H1N1)pdm09 strain. Sera collected pre-vaccination, post-vaccination and post-challenge were analyzed for antibody-mediated inhibition of NA activity using a recombinant H7N1_CA09_ virus. Antigenic sites were further identified with linear and conformational peptide microarrays spanning the full NA of A/California/04/2009(H1N1)pdm09. Vaccine-induced NA-specific antibodies inhibited the enzymatic function of NA in both animal models. The antibodies target critical sites of NA such as the enzymatic site, second sialic binding site and framework residues, shown here by high-resolution epitope mapping. New possible antigenic sites were identified that potentially block the catalytic activity of NA, including an epitope recognized solely in pigs and ferrets with neuraminidase inhibition, which could be a key antigenic site affecting NA function. These findings show that our influenza DNA vaccine candidate induces NA-specific antibodies that target known critical sites, and new potential antigenic sites of NA, inhibiting the catalytic activity of NA.

## 1 Introduction

Influenza continues to be a serious health concern worldwide causing an estimated 3-5 million cases of severe disease and 290,000 - 650,000 deaths annually (1). The seasonal influenza vaccines reduce morbidity and mortality; however, they are limited by time-consuming design, the requirement for yearly prediction of circulating virus strains, and short-term immunity (2–4). Moreover, the seasonal influenza vaccines provide little protection against potential pandemic viruses originating from animals (5). Therefore, a need for continued rational development of novel vaccine strategies to replace the current seasonal vaccines remains.

Current vaccine efforts predominantly focus on hemagglutinin (HA) (6–8), which is the most abundant surface protein of the influenza virus. Antibodies targeting HA can prevent attachment of the virus to terminal sialic acids on host cells or membrane fusion and thereby block or neutralize viral infection. However, there is merit in targeting the second major surface protein, neuraminidase (NA). NA accounts for approximately 10-20% of the total glycoproteins on the virion surface and assembles as a homotetramer consisting of monomers of approximately 470 amino acids (9). The best characterized function of NA is its enzymatic activity that cleaves terminal sialic acids on glycans expressed on the host cell surface, which enables the release of new virions from the infected cell (9). The active site contains eight highly conserved residues that directly interact with sialic acids that are Arg-118, Asp-151, Arg-152, Arg-224, Glu-276, Arg-292, Arg-371 and Tyr-406 (9). In addition to the active site, there is a second sialic binding site where sialic acids interact with residues Ser-367, Ser-370, Ser-372, Asn-400, Trp-403 and Lys-432. The ten framework residues that include Glu-119, Arg-156, Trp-178, Ser-179, Asp-198, Ile-222, Glu-227, Glu-277, Asn-294 and Glu-425, do not directly interact with sialic acids, but serve an important structural role of the NA protein (9). Besides its involvement in virus release, NA may also play a role in virus entry. Mucins, which are sialylated glycoproteins that form the mucus in airways, are cleaved by NA hence enabling the influenza virus to move through the mucus to establish an infection (9–12).

Vaccine-induced antibodies should ideally target key viral functions to protect against infection and neutralizing antibodies are often a correlate of vaccine efficacy (13). Although NA-specific antibodies do not provide neutralizing immunity, antibodies targeting NA have an impact on the severity of the infection by preventing the release and spread of new virions; thus, limiting an established infection (12, 13). Several studies have reported an association between NA-inhibiting antibodies and reduced influenza virus shedding and disease severity (14–17). This critical feature of NA in viral infection and release makes it an attractive target for prophylactic and therapeutic drugs and highlights the important role of NA immunity (18, 19).

We developed an influenza DNA vaccine that encodes surface and internal proteins from pandemic H1N1 and H3N2 influenza viruses (20–23), including the NA from the pandemic A/California/04/2009(H1N1)pdm09 virus. The vaccine confers protection in both ferrets and pigs against a homologous challenge with A/California/7/2009(H1N1)pdm09 as well as against a lethal challenge in ferrets with a highly pathogenic avian A/Viet Nam/1203/2004(H5N1), highlighting the broadness of the vaccine-induced immune response (22, 23). The DNA vaccine does not induce sterilizing immunity; however, elicits anamnestic immune responses. Following viral challenge, the animals receiving the DNA vaccine had a shorter duration of viral shedding and less severe disease symptoms compared to control animals (22, 23). The observed vaccine protection is likely conferred by multiple components in the vaccine. This study defines vaccine-induced NA antibody responses, including antibody-binding sites and functional responses to N1 of the pandemic influenza virus A/California/04/2009(H1N1)pdm09 in DNA vaccinated and virus-challenged ferrets and pigs.

## 2 Materials and methods

### 2.1 Influenza A DNA vaccine

The DNA vaccine comprises six plasmids that encode surface and internal proteins of H1N1 and H3N2 influenza viruses (20, 21). Briefly, the sequences were derived from pandemic influenza strains that emerged in 1918 (H1N1), 1968 (H3N2) and 2009 (H1N1). The encoded proteins include the HA and NA proteins of A/California/04/2009(H1N1)pdm09, the HA and NA of A/Aichi/2/1968(H3N2), the nucleoprotein of A/Brevig Mission/1/18(H1N1), and the matrix proteins of A/Brevig Mission/1/18(H1N1). The Genbank protein accession numbers are ACP41105, ACP41107, BAF48361.1, BAF48362.1, AAV48837.1, AAN06598.1, and AAN06597.1, respectively, and corresponding codon optimized sequences can be found in patent WO2010060430A2. The gene sequences were human codon optimized, except for the M gene, where post-transcriptional splicing is required for production of M1 and M2 (20–23). The influenza protein encoding fragments were made synthetically with the appropriate restriction enzyme sites and Kozak sequence (GCCACC) -1 base upstream of the start codon, for efficient cloning and translation. The minimal NTC9385R plasmid (Nature Technology Corporation, USA), free of antibiotic resistance genes, was used as the expression vector backbone (24). The DNA vaccine vector map is described in detail elsewhere; all genes were cloned into the vector upstream of the polyadenylation site (24). Plasmids are produced and isolated in the HyperGRO™ fermentation process at Nature Technology Corporation (25). The specification for endotoxin levels of plasmid preparations in this process is <100 EU/mg using the Limulus Amebocyte Lysate assay. Endotoxin levels are not available for the vaccines used in the below mentioned animal studies; however, other preparations from this production method yielded endotoxin levels below 2 EU/mg (26). The six plasmids were mixed at equal weight ratios with Phosphate Buffered Saline (PBS) and subsequently mixed 1:1 (pigs) or 1:3 (ferrets) with α-tocopherol-based aqueous solution (Diluvac Forte^®^, MSD Vet Animal Health) as an adjuvanted vehicle (22, 23).

### 2.2 Pig study

**Figure 1:**
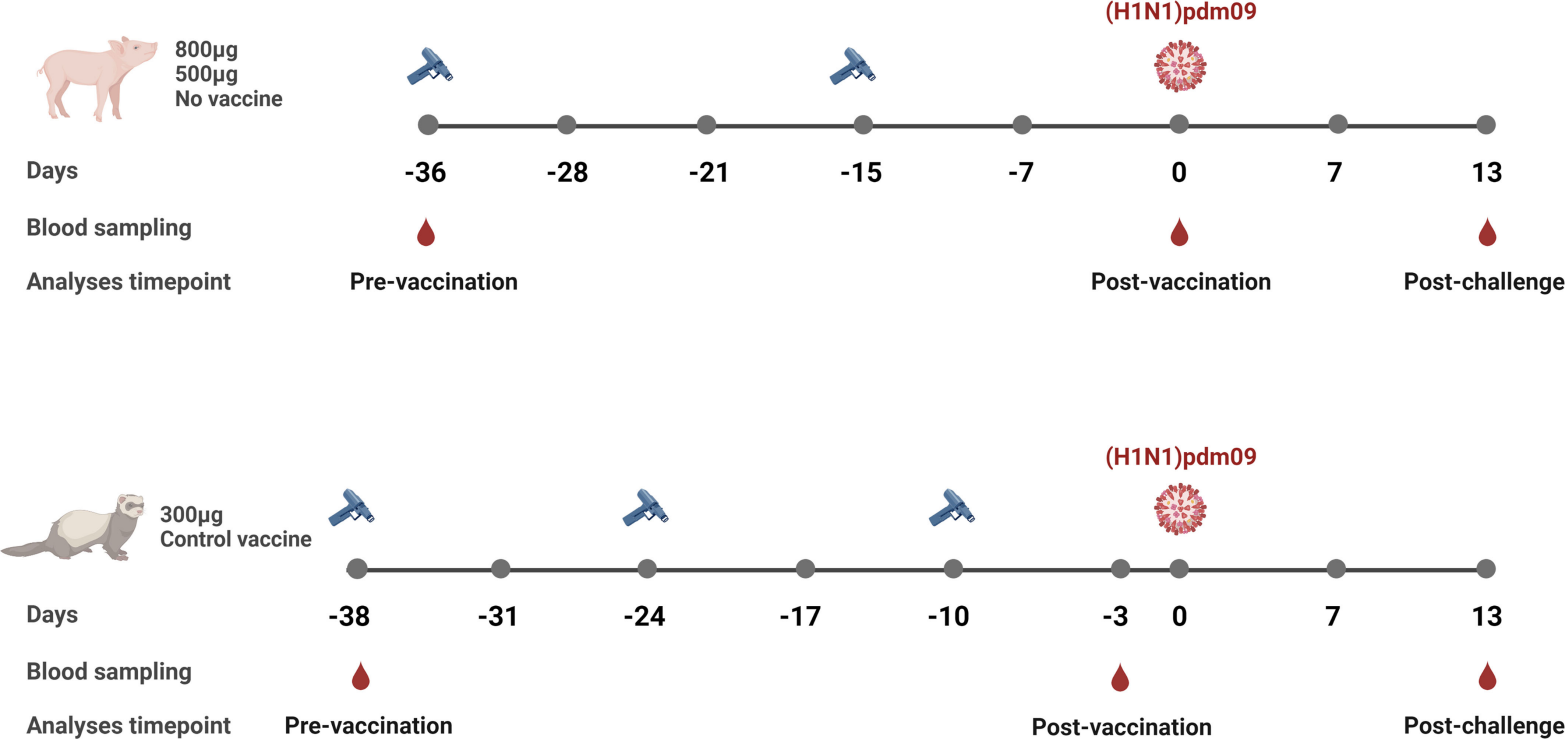
Overview of the influenza DNA vaccinated pig and ferret studies. Pigs were vaccinated twice (day -36 and day -15), 3 weeks apart with either 800 µg or 500 µg per vaccination of the influenza DNA vaccine. Unvaccinated pigs served as a control group. Serum samples were collected prior to first vaccination (day -36), 2 weeks after the second vaccination (day 0), and 2 weeks post-challenge (day 13). Ferrets were vaccinated three times (day -38, -24 and -10), 2 weeks apart with 300 µg with the same influenza DNA vaccine. The control group received 300 µg per vaccination of the vaccine vector encoding an unrelated Zika virus envelope glycoprotein. Serum samples were collected prior to first vaccination (day -38), 1 week after the third vaccination (day -3), and 2 weeks post-challenge (day 13). All animals were challenged with A/California/7/2009(H1N1)pdm09. Created with BioRender.com.

Fifteen five-to-six-week-old, recently weaned, male pigs were randomly assigned to three groups (n=5/group); 1) 500 µg of DNA vaccine, 2) 800 µg of DNA vaccine and 3) unvaccinated control group (**Figure 1**) (22). With an interval of three weeks, the animals were vaccinated twice on the dorsal side of the back using the needle-free Intra-Dermal Application of Liquids device (IDAL^®^ MSD Animal Health) (27). Two weeks after the second immunization, each pig was challenged intranasally with 10^6^ 50% Tissue Culture Infectious Dose (TCID_50_) of pandemic A/California/7/2009 (H1N1)pdm09. Clinical signs of disease, adverse effects related to vaccination, rectal body temperatures and clinical scores of infection have been published elsewhere in detail (22). Serum samples were collected on day -36, -28, -21, -15, -7, 0, 7 and 13 post-challenge. The study was terminated day 13 post-challenge. The study was approved by the Ethics Committee for Animal Experimentation from the Institute of Agrifood Research and Technology and the Animal Experimentation Commission from the Autonomous Community of Catalonia Government in compliance with the Directive, UE 63/2010 and the Spanish Legislation, RD53/2013 and the Catalan Law 5/1995 and Decree 214/1997.

### 2.3 Ferret study

Four and a half to six months old male ferrets were assigned to two experimental groups (n=5/group) (**Figure 1**) (23). The first group received 300 µg of the influenza DNA vaccine while the control group received 300 µg of a DNA vaccine encoding an unrelated Zika virus envelope glycoprotein. Both vaccines were expressed from the same plasmid vector (NTC9385R, Nature Technologies Corporation, USA) and formulated in Diluvac Forte^®^ (MSD Vet Animal Health). The animals were vaccinated on day -38, -24 and -10, using the needle-free Intra-Dermal Application of Liquids (IDAL^®^ MSD Animal Health) device (27). Ten days after the last immunization, animals were challenged intranasally with 10^6^ 50% Egg Infectivity Dose of A/California/7/2009(H1N1)pdm09. The animals were monitored daily for weight, temperature and clinical signs of disease or any adverse vaccination-related effects and have been reported in detail elsewhere (23). Whole blood samples were collected prior to the first immunization (day -38), post last immunization (day-3) and post challenge (day 13). All husbandry and procedural work was conducted by NIBSC animal technicians in accordance with UK Home Office license numbers 80/2537 and PCB39ED3C, under Protocol numbers 4 and 5, respectively (23).

### 2.4 Enzyme-linked immunosorbent assay (ELISA)

NA-specific immunoglobulin G (IgG) titers were measured using ELISA. Nunc™ MaxiSorp™ plates were coated with 100 µl of 2 U/ml active NA protein (Influenza A H1N1 A/California/04/2009) (Sino Biological, USA) and incubated with blocking buffer (SSI Dilution Buffer [SSI Diagnostica], + 2% skim milk powder) for one hour. Washing (PBS (1.5 mM KH_2_PO_4_, 8 mM Na_2_HPO_4_, 137 mM NaCl, and 2.7 mM KCl; pH = 7.4; PBS) + 1% Triton X100) was done between each incubation step. Plates were incubated with a 4-fold serial dilution of pig sera or a 5-fold serial dilution of ferret sera for one hour. Horseradish peroxidase conjugated goat-anti-pig IgG antibody (BioRad, USA) or goat-anti-ferret IgG antibody (Novus Biologicals, USA) was added and incubated for one hour. All incubations were performed on an orbital shaker. BM Chemiluminescent Substrate (Sigma-Aldrich, USA) was added for ten minutes and luminescence read-out was measured using a FLUOstar Omega microplate reader (BMG Labtech, Germany).

### 2.5 Viruses used for neuraminidase inhibition assays

The reassortant H7N1_CA09_ (NIBRG-127) virus strain was generated by reverse genetics and produced by the National Institute for Biological Standards and Control (NIBSC, United Kingdom). The NA sequence genes were derived from A/California/7/09(H1N1)pdm09, the HA was derived from A/Equine/Prague/56(H7N7) and the remaining segments from A/Puerto Rico/8/34 (H1N1) (NIBSC, UK) (23).

### 2.6 Enzyme-linked lectin assay (ELLA)

The NA inhibition (NI) assay ELLA was performed as described with minor modifications (23, 28). In brief, 4-fold serial dilutions of heat-inactivated sera were incubated with virus for 60 minutes at room temperature, transferred to microtitre plates coated with 2.5 µg fetuin (Bio-Rad, USA) and incubated for 18 hours at 37°C. Plates were washed with PBS containing 0.05% Tween20 (PBST) and subsequently incubated with 0.1 µg peanut agglutinin conjugated to horse-radish peroxidase (PNA-HRP, Sigma-Aldrich, USA) for two hours at room temperature. Plates were washed in PBST and incubated for 10 minutes with BM Chemiluminescent Substrate (Sigma-Aldrich, USA). Luminescence was measured using a FLUOstar Omega microplate reader (BMG Labtech, Germany).

### 2.7 Fluorescence-based neuraminidase inhibition assay (MUNANA method)

Antibody-mediated NI activity was measured using the NA-Fluor Influenza Neuraminidase Assay kit (Life Technologies, USA) according to the manufacturer’s protocol. Briefly, 4-fold serial dilutions of sera were incubated with virus for 30 minutes at 37°C in microtitre plates as previously described (22). NA-Fluor™ Substrate was added and plates were incubated for one hour at 37°C protected from light before addition of NA-Fluor™ Stop Solution. Fluorescence was measured using a FLUOstar Omega microplate reader (BMG Labtech, Germany).

### 2.8 Solid phase peptide synthesis

The linear peptide microarray comprised overlapping 20 amino acid peptides with a 10 amino acid overlap of the complete NA protein of A/California/04/2009(H1N1)pdm09. Peptides were prepared by automated peptide synthesis on a Syro II peptide synthesizer (MultiSynTech, Witten, Germany) by standard solid-phase peptide synthesis (SPPS) on 6 mg (1.4 µmol) TentaGel S Rink Amide resin (loading 0.24 mmol/g) with Fmoc for protection of Nα-amino groups in a reaction block holding 96 tips (0.2 mL). Side-chain protecting groups were tert-butyl (Ser, Thr), 2,2,4,6,7-pentamethyl-dihydrobenzofuran-5-sulfonyl (Pbf, for Arg), Boc (Lys), and trityl (Trt, for Asn, Gln, His). Nα-Fmoc amino acids (4.0 equiv) were coupled using HBTU (3.9 equiv), 1-HOBt (4.0 equiv), and DIPEA (9.8 equiv) in NMP for 120 min. Followed by a washing procedure consisting of three thorough washes with NMP, one wash with DCM followed by another three washes with NMP. Capping of the unreacted N-terminal amines was performed following each coupling step using three consecutive treatments with acetic anhydride−DCM solution (1:4) of 15 min each and thorough NMP washing was undertaken between treatments. Nα-Fmoc deprotection was performed using piperidine−DMF (2:3) for 3 min followed by piperidine−DMF (1:4) for 12 min. The peptides and glycopeptides were released from the solid support, and all acid-labile protecting groups were removed by treatment with TFA/TES/H2O (95:2:3) for 2 h. The TFA solutions were concentrated by nitrogen flow, the compounds were precipitated with Et2O, and crude materials were collected as white pellets after centrifugation.

### 2.9 Microarray printing

The peptides were diluted 20-fold in print buffer (17 mM monosodium phosphate (NaH_2_PO_4_), 133 mM disodium phosphate (Na_2_HPO_4_), and 0.03% Sodium azide (NaN_3_); pH 8.5). 35 uL of each peptide solution was distributed into a 384 well source plate (Corning, USA). The peptides were immobilized on N-hydroxysuccinimide (NHS) activated hydrogel-coated MPX16 glass slides (Schott Nexterion, Slide H) using a BioRobotics MicroGrid II spotter (Genomics Solution Ltd) with Stealth 3B Microspotting pins (ArrayIt). A 4-pin configuration was used to print the peptides into 16 subarrays per slide. Each linear peptide was printed in triplicate. The deposit volume was approximately 6 nL per spot. After printing, the microarrays were incubated at a 70% humidity for one hour. The remaining NHS groups were deactivated with blocking buffer (50 mM ethanolamine in 50 mM borate buffer; pH 9.2) for one hour and the microarray slides were rinsed with deionized water and spun dry. The blocked glass slides were fitted into superstructures; 2/16/48 well (FAST FRAME, Schleicher & Schuell (Whatman)) to make separate identical peptide libraries.

### 2.10 Linear peptide microarray assay

Pools of sera from each animal study were screened on the linear peptide microarray. Sera were diluted 20-fold in PLiP buffer (0.5 M NaCl, 3 mM KCl, 1.5 mM, KH_2_PO_4_, 6.5 mM Na_2_HPO_4_, pH 7.4, 3% BSA 1% Triton-X 100, pH 7.4), and 100 μL was added to each well on the peptide microarray and incubated overnight on a shaking plate with a slow rotation. Microarrays were washed three times with PBS (1.5 mM KH_2_PO_4_, 8 mM Na_2_HPO_4_, 137 mM NaCl, and 2.7 mM KCl; pH = 7.4) and samples were subsequently incubated for one hour with a biotinylated anti-swine IgG secondary antibody (Sigma-Aldrich, USA) diluted 500-fold in PLiP buffer. Microarrays were washed and incubated for one hour with streptavidin Alexa Fluor 647 conjugate (Thermo Fisher Scientific, USA), diluted 500-fold in PLiP buffer. Microarrays were washed, rinsed with deionized water and dried by centrifugation. All wash steps were preformed three times for five minutes in a humidified chamber at room temperature, with shaking. Slides were scanned using a ProScanArray microarray scanner (Perkin Elmer) and images were analyzed with Scan Array Express software. Spots were identified using automated spot finding with manual adjustments for irregularities in print. The final data was obtained from the mean spot Relative Fluorescence Units (RFU) from all replicate spots for each sample. Spot intensities were determined by subtracting the median pixel intensity of the local background from the average pixel intensity within the spot. The quality control covered intra- and interchip quality analysis of replicates. For the selected peptides, serum samples with relative fluorescent values higher than two standard deviations over the mean of the control group were designated as positive.

### 2.11 Peptide microarray with cyclic constrained peptides (conformational)

Customized cyclic constrained peptide microarrays (PEPperPRINT GmdH, Germany) covering the NA of A/California/04/2009(H1N1)pdm09 (GenBank accession no. ACP41107.1) were used to identify conformational epitopes in pooled sera and individual animals. The peptide array comprised overlapping peptides of 13 amino acid residues with an eight amino acid overlap. The experiment was performed according to the manufacturer’s protocol. In brief, the peptide microarrays were hydrated for 15 minutes with washing buffer (PBS with 0.005% Tween20, pH 7.4) followed by a 30 minutes incubation with Rockland Blocking Buffer MB-070 (Rockland, USA). All wash and incubation steps were performed on an orbital shaker at 140 rpm. The peptide arrays were pre-stained with either a 1:500 dilution of anti-Swine IgG (H+L)-Biotin (Rockland, USA) or a 1:200 dilution of anti-Ferret IgG (γ-chain specific)-Rhodamine (Sigma-Aldrich, USA) secondary antibody and scanned to identify any unspecific interactions with peptides. Microarrays were equilibrated 15 minutes in staining buffer (washing buffer with 10% Rockland Blocking Buffer) before adding the serum samples. Samples were diluted 20-fold for pigs and 10-fold for ferrets in staining buffer and incubated overnight at 4˚C. Microarrays were washed twice for 10 seconds in washing buffer and incubated one hour with anti-Swine IgG (H+L)-Biotin (Sigma-Aldrich, USA) for pig sera or anti-Ferret IgG (γ-chain specific)-Rhodamine (Sigma-Aldrich, USA) for ferret sera. For the pig sera microarray, an additional 45 minute incubation step with 1:500 diluted streptavidin Alexa Fluor 647 conjugate (Thermo Fisher Scientific, USA) was performed. Microarrays were subsequently washed, dipped in 1 mM Tris, pH 7.4 and dried by centrifugation at 1000 rpm for one minute. The microarrays were scanned on an Agilent SureScan microarray scanner (Agilent Scientific Instruments) and analyzed using MAPIX Analyzer Software (Innopsys, France).

### 2.12 Computational and statistical analysis

The NI titer is defined as the reciprocal of the serum dilution that inhibited 50% of NA activity calculated from a 4-parameter logistic regression curve. NI titers were normalized according to a positive control included on each plate. The fold change in serum antibody binding on the microarrays was calculated by dividing the fluorescent intensity measured for a specific peptide post-immunization or post-challenge with the fluorescent intensity measured for the same peptide pre-immunization. A fold change cutoff was defined as the mean fold change + SD multiplied with a 99% confidence level coefficient (*f*) according to the number of peptides (triplicates) as described in (29). B-cell linear and discontinuous epitope predictions were done using BepiPred (https://services.healthtech.dtu.dk/service.php?BepiPred-2.0) and DiscoTope (https://services.healthtech.dtu.dk/service.php?DiscoTope-2.0), respectively. For BepiPred, the epitope threshold was set at 0.6 for increased stringency (specificity: 0.95; sensitivity: 0.10). All data were tested for normal distribution using the Shapiro-Wilk normality test. Correlation of serological parameters was performed using Spearman’s rank correlation test for non-normally distributed data and Pearson correlation test for normally distributed data. For analysis of continuous data (time points) differences were tested using the Friedman’s test. Comparison of animal groups was done using a *t-*test for normally distributed data and Mann-Whitney test was used for non-normally distributed data. A p-value < 0.05 was considered significant. All statistical analyses were performed in GraphPad Prism^®^ version 8.3.0 (GraphPad software, USA).

## 3 Results

### 3.1 The influenza DNA vaccine efficiently induces NA-specific antibody responses in different animal species

**Figure 2:**
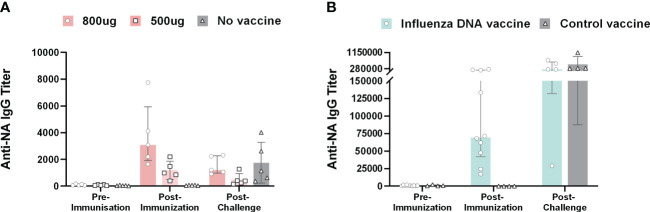
Anti-NA antibody responses in pigs and ferrets after influenza DNA vaccination and subsequent homologous virus challenge. **(A)** Anti-NA IgG end-point titers in pigs immunized with 800 µg (dark pink), 500 µg (light pink) influenza DNA vaccine and unvaccinated pigs (grey). **(B)** Anti-NA IgG end-point titers for ferrets immunized with influenza DNA vaccine (pale green) and ferrets immunized with a control vaccine (grey). Bar graphs indicate median with interquartile range.

In all vaccinated pigs, the DNA vaccine elicited NA-specific IgG responses post-immunization measured by ELISA (**Figure 2**). The two vaccine doses induced antibody responses in a dose-dependent manner. The median end-point titers after the second vaccine dose were 3074 (range: 1644 – 7748) and 985 (range: 381 – 2184) for pigs that received 800 µg and 500 µg DNA, respectively. The level of NA-specific IgG titers post-immunization did not differ significantly between the two vaccine dose groups. Post-challenge, the median anti-NA IgG end-point titer was approximately three fold lower compared to post-vaccination at 1214 (range: 904 – 2313) and 389 (range: 150 – 1271) for pigs vaccinated with 800 µg and 500 µg DNA, respectively. Unvaccinated animals only developed antibodies specific for NA post-challenge with a median titer of 1147 (range: 374 – 4007). Likewise, ferrets immunized with the influenza DNA vaccine developed NA-specific IgG responses with a median end-point titer of 69551 (range 17116 – 239072). Ferrets vaccinated with a control DNA vaccine had detectable NA antibodies after viral challenge.

### 3.2 Vaccine-induced antibodies differentially inhibit NA activity in two assays

**Figure 3:**
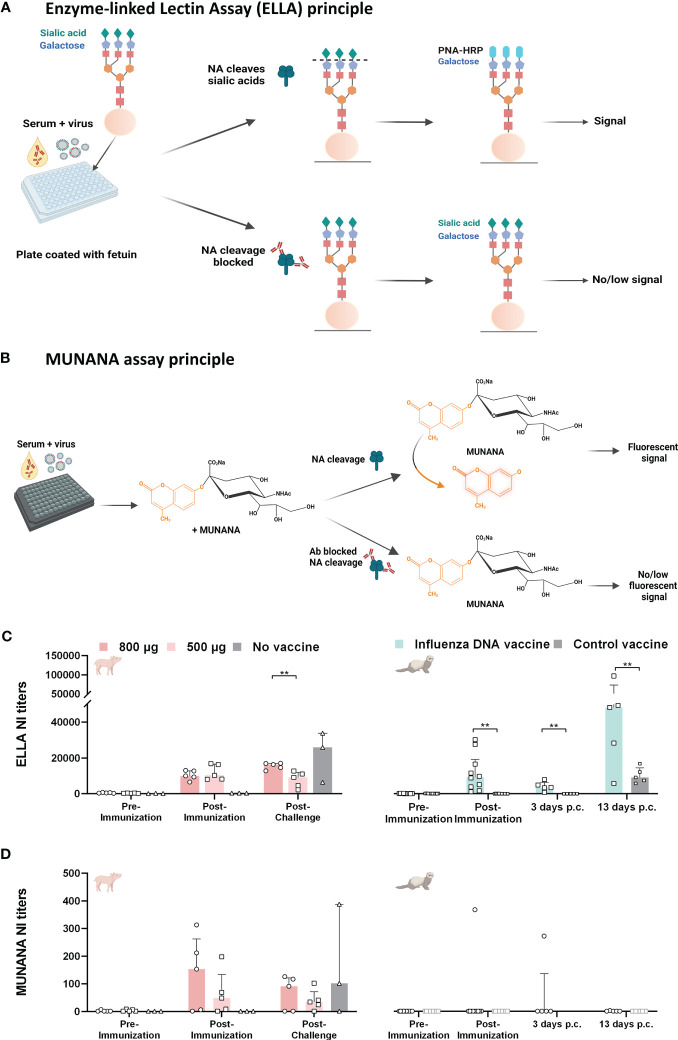
NA inhibition activity in pigs and ferrets after influenza DNA vaccination and subsequent homologous virus challenge. **(A)** The assay principle of the ELLA neuraminidase inhibition assay. In brief, serum and influenza virus are added to a plate coated with fetuin protein. Antibodies that bind and block NA cleavage of sialic acids on the fetuin protein will result in no/low signal, leaving the protein intact. However, antibodies that fail to block NA cleavage of sialic acids enables the binding of peanut agglutinin conjugated to horse-radish peroxidase (PNA-HRP) to exposed galactose molecules, resulting in a detectable signal. **(B)** The assay principle of the MUNANA neuraminidase inhibition assay. In brief, serum and influenza virus are added to a plate, followed by the addition of a small, synthetic substrate MUNANA (4-(methylumbelliferyl)-N-acetylneuraminic acid). Similar to the ELLA principle, antibodies that block NA cleavage of the MUNANA substrate, leaves the substrate intact and no fluorescent signal is detected. If the antibodies are not able to block NA cleavage of the substrate, the NA protein cleaves the substrate and will give a fluorescent signal. **(C)** NI titers measured by ELLA for pigs immunized with 800 µg DNA (dark pink), 500 µg DNA (light pink) and unvaccinated pigs (grey), and ferrets immunized with influenza DNA vaccine (pale green) and control vaccinated ferrets (grey). ELLA NI titers for ferrets are reprinted from Vaccine, Volume 39, Issue 34, Kate Guilfoyle, Diane Major, Sarah Skeldon, Heather James, Jeanette L Tingstedt, Charlotta Polacek, Ria Lassauniére, Othmar G Engelhardt, Anders Fomsgaard, Protective efficacy of a polyvalent influenza A DNA vaccine against both homologous (H1N1pdm09) and heterologous (H5N1) challenge in the ferret model, pages 4903-4913, 9 August 2021, with permission from Elsevier [23]. **(D)** NI antibody titers measured with the MUNANA method for the same groups of animals as in **(C)**. Color coding as in **(C)** It should be noted that for the ferrets, five animals were euthanized 3 days post-challenge (p.c.), this subset included the only ferret (ferret 23) that displayed NA inhibition in MUNANA. Bar graphs indicate median with interquartile range. **(A, B)** Created with BioRender.com.

NA inhibition (NI) was measured using two different methods, the ELLA and the MUNANA method. The ELLA method measures inhibition of neuraminidase cleavage of sialic acids on immobilized complex carbohydrates on fetuin, a 48.4 kDa glycoprotein (**Figure 3A**). Conversely, the MUNANA method measures the inhibition of neuraminidase cleavage of a small, soluble substrate (**Figure 3B**).

Since antibodies targeting wildtype HA protein may block NA activity through steric hindrance, thus interfere with measuring specific NA inhibition (NI) (30–32), we used a reassortant influenza virus, H7N1_CA09_ (NIBRG-127), containing NA from A/California/04/2009(H1N1)pdm09 and a distant HA from A/Equine/Prague/56(H7N7) to measure N1-specific activity in both assays.

In all immunized pigs, the influenza DNA vaccine elicited functional antibodies capable of inhibiting NA activity post-immunization when measured in the ELLA assay (**Figures 3B, C**). Pigs that received 800 µg DNA vaccine had a significant boost of NI antibody titers after challenge. Post-challenge, NI titers were higher in pigs immunized with the highest dose (median titer: 16322; range: 12789 – 17035) compared to pigs receiving the low dose DNA vaccination (median titer: 9104; range: 2234 – 12721; P = 0.0074) (**Figure 3C**). Conversely, NI activity measured by the MUNANA method did not differ significantly between the vaccinated pig groups post-immunization and post-challenge (**Figure 3D**). The NI titers measured by either assay did not correlate with NA-specific IgG titers in vaccinated pigs (ELLA: Spearman r = 0.4897, P = 0.0909; MUNANA: Spearman r = 0.4567, P = 0.1173).

Despite the presence of NA-specific antibodies measured by ELISA and NI activity measured by ELLA [reported in a previous study (23)] (**Figure 3C**), vaccinated ferrets had no detectable NI activity when measured by the MUNANA method, with the exception of one animal (**Figure 3D**). In contrast to that observed in the pigs, NI activity measured by ELLA directly correlated with NA-specific IgG titers post-immunization in ferrets (Pearson r = 0.8620, P = 0.001). The lack of detectable NI activity in the MUNANA method precluded a correlation analysis with binding IgG titers for ferrets. Taken together, the differences between pigs and ferrets in NI titers relative to NA-specific binding IgG titers and differential NI reactivity in the two different assays suggest that factors other than antibody amount determine NI activity. We next sought to identify antigenic regions on the NA protein.

### 3.3 Interspecies differences in recognition of linear and conformational B cell epitopes post-vaccination

**Figure 4:**
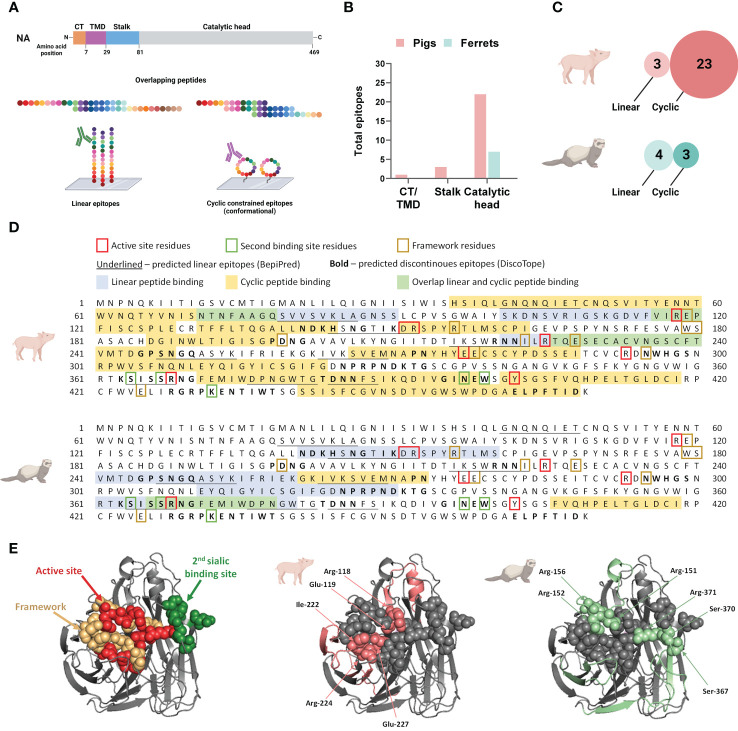
Neuraminidase B cell epitopes targeted following influenza DNA vaccination in pigs and ferrets. **(A)** Post-vaccination serum pools for each species was tested on both a linear and cyclic constrained peptide array. **(B)** Distribution of overall antibody targets post vaccination according to the NA protein domains: cytoplasmic tail (CT), transmembrane domain (TMD), stalk, and catalytic head. **(C)** Relative number of peptides targeted on the two peptide array formats by antibodies in post-vaccination sera in pigs and ferrets. **(D)** The NA protein sequence of A/California/04/2009(H1N1)pdm09 showing the peptide sequences recognized by antibodies on the linear and cyclic constrained peptide microarray relative to functional and structural critical amino acid residues and predicted linear B cell epitopes defined by BepiPred as well as predicted discontinuous B cell epitopes predicted by DiscoTope. **(E)** Crystal structure of a A/California/04/2009(H1N1)pdm09 NA monomer (PDB ID:3NSS) indicating the active site, framework residues, and second sialic acid binding site followed by regions targeted by serum antibodies in pigs and ferrets on the linear peptide microarray. **(A, C)** Created with BioRender.com.

To define NA-specific B cell epitopes targeted after DNA vaccination and subsequent vaccine homologous virus challenge, we tested pooled serum samples from pigs and ferrets at three time points: pre-vaccination, post-vaccination and post-challenge. Antibody binding profiles in the serum pools were assessed with peptide microarrays printed with overlapping linear or cyclic constrained peptides (**Figure 4A**). Antibody targeting of a peptide was considered positive when the fluorescence intensity exceeded a 99% confidence level cut-off value calculated from species-specific control data post-vaccination. The presence of maternal antibodies in pigs necessitated separate cut-off values for pigs and ferrets.

Post-vaccination, the majority of antibodies targeted the catalytic head, which is the larger and more accessible part of this surface protein (**Figure 4B**). When considering the combined number of peptides targeted by both the linear and cyclic constrained peptide microarrays, pig serum antibodies targeted more peptides compared to ferrets with substantial differences between the array types for the two species. Post-vaccination, pig sera targeted fewer peptides on the linear peptide array (n = 3) than on the cyclic constrained peptide array (n = 23) while the ferret sera targeted an almost equal number of peptides on both arrays with substantially less peptides on the cyclic constrained peptide array compared to the pigs (linear: n = 4; cyclic constrained: n = 3; **Figure 4C**). In some instances, there were parallel detection of the same amino acid sequence on the two arrays (**Figure 4D**). For the pig sera, all three linear peptides overlapped partially or completely with peptides targeted on the cyclic constrained peptide array. In contrast, for the ferret sera, only one of the four peptides identified on the linear peptide array overlapped with a peptide identified on the cyclic constrained peptide array. Also, several targeted epitopes overlapped with *in silico* predicted linear and discontinuous epitopes (**Figure 4D**).

Despite receiving the same DNA vaccine, pig and ferret sera targeted different sites on the NA protein and with different coverage (**Figures 4D, E**). Overall, pig sera targeted epitopes more broadly across the NA protein including 6 of 8 active site residues, 1 of 5 second binding site residues, and 5 of 10 framework residues. In comparison, the ferret sera targeted fewer epitopes, comprising 3 of the 8 active site residues, 2 of 5 second binding site residues, and 1 of 10 framework residues. The narrower coverage of critical functional and structural residues targeted by ferrets may contribute to the observed functional differences observed between pig and ferret sera in the MUNANA assay (**Figure 3**).

### 3.4 B cell epitopes targeted after a vaccine homologous influenza H1N1 viral challenge

**Figure 5:**
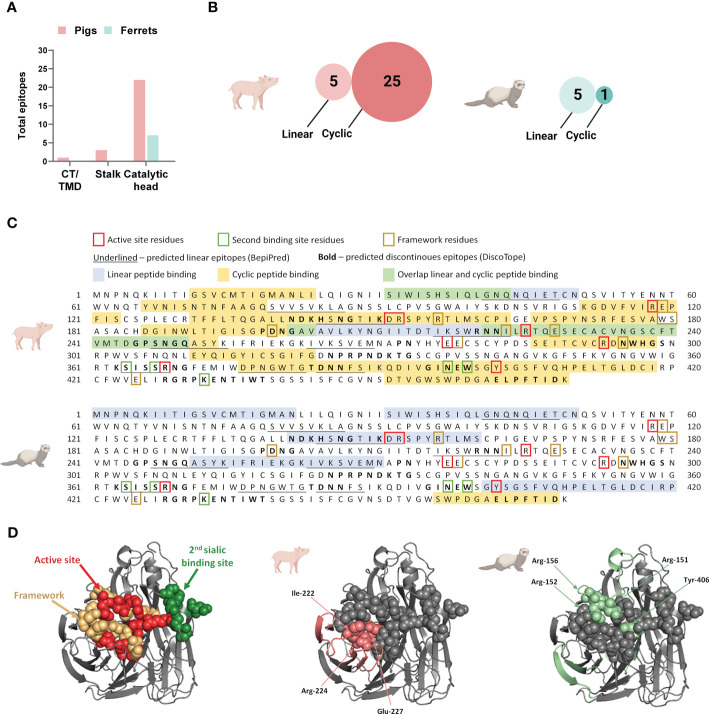
Neuraminidase B cell epitopes targeted following A/California/7/2009(H1N1)pdm09 influenza virus challenge in DNA vaccinated pigs and ferrets. **(A)** Distribution of antibody targets according to NA protein domains: cytoplasmic tail (CT), transmembrane domain (TMD), stalk, and catalytic head. **(B)** Relative number of peptides targeted on the two peptide array formats by antibodies in post-challenge sera in pigs and ferrets. **(C)** The NA protein sequence of A/California/04/2009 (H1N1pdm09) showing the peptide sequences recognized by antibodies on the linear and cyclic constrained peptide microarray relative the functional and structural critical amino acid residues and predicted linear B cell epitopes defined by BepiPred as well as predicted discontinuous B cell epitopes predicted by DiscoTope. **(D)** Crystal structure of a A/California/04/2009(H1N1)pdm09 NA monomer (PDB ID:3NSS) indicating the active site, framework residues, and second sialic acid binding site followed by regions targeted by serum antibodies in pigs and ferrets on the linear peptide microarray. **(B)** Created with BioRender.com.

Post-challenge, pig and ferret anti-NA antibodies continued to target primarily the catalytic head, although ferret antibodies targeted additional regions at the N-terminus (**Figures 5A, C**). Similar to that observed post-vaccination, pig sera more readily targeted cyclic constrained peptides compared to linear peptides (25 vs. 5), while ferret serum antibody binding to cyclic constrained peptides remained limited (**Figure 5B**).

Overall, the viral challenge induced some changes in peptide recognition from post-vaccination. Post-challenge, pig serum antibodies targeted 30 peptides on both arrays combined, that included 15 peptides targeted post-vaccination and 15 newly identified peptides. Peptides targeted both post-vaccination and post-challenge include a linear peptide spanning amino acid 221−240 and cyclic constrained peptides (individual or overlapping) spanning amino acids 36-43, 66−78, 116-123, 131−143, 151−163, 186−198, 226−248, 311−323, 376−398, 401−418, and 451−468 (**Figure 5C**). Additional regions targeted post-challenge include peptides spanning amino acids 11−23, 31-35, 144-150, 166−178, 199−220, 249-253 and 289−298. Post-challenge serum antibodies attained a modest increase in coverage of critical functional or structural residues on the NA protein (**Figures 5C, D**). Post-challenge, the pig sera targeted 6 of the 8 active site residues, 2 of the 5 second binding site residues, and 6 of 10 framework residues.

In contrast, viral challenge did not increase the number of ferret antibodies targeting critical functional or structural residues on the NA protein (**Figures 5C, D**). Post-challenge, the ferret sera targeted 3 of the 8 active site residues, none of the 5 second binding site residues, and 1 of 10 framework residues. Both post-vaccination and post-challenge, the ferret sera consistently targeted two regions on the NA protein that include amino acids 141−160 and 251−270 (**Figures 4C**, **5C**). Post-challenge, the sera targeted three additional regions that include the transmembrane domain amino acids 1−20, the stalk amino acids 31−50, and the C-terminus amino acids 456−468 (**Figure 5C**). Conversely, two regions targeted post-vaccination were lost post-challenge, including amino acids 311−330 and 361−380.

### 3.5 Identification of a novel epitope potentially targeted by antibodies with neuraminidase inhibition activity

**Figure 6:**
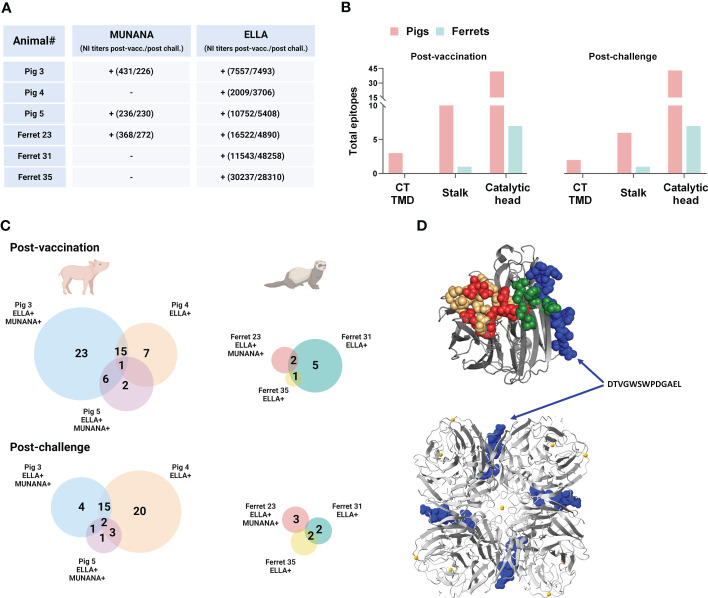
Neuraminidase B cell epitopes targeted in DNA vaccinated pigs and ferrets with and without NA inhibition (NI) in ELLA and MUNANA. **(A)** Scheme of individual pigs and ferrets with (+) and without **(-)** NI in ELLA and MUNANA. NI titers post-vaccination and post-challenge are denoted in brackets for each individual animal. **(B)** Distribution of antibody targets according to NA protein domains: cytoplasmic tail (CT), transmembrane domain (TMD), stalk, and catalytic head. **(C)** Venn diagrams showing the total number of epitopes recognized in pigs and ferrets post-vaccination and post-challenge. Shared epitopes between two or more animals are stated for each animal species. NI in ELLA and/or MUNANA is indicated for each animal with a (+). **(D)** Crystal structure of a A/California/04/2009(H1N1)pdm09 NA monomer (top) and tetramer (bottom) (PDB ID:3NSS) showing the location of the epitope DTVGWSWPDGAEL (in blue), only recognized in animals with NI in both ELLA and MUNANA. **(A, C) (D)** was created in YASARA Structure using the MUSTANG algorithm. Created with BioRender.com.

The observed species-specific differences in antibody binding may, in part, explain the differential detection of serum antibody-mediated neuraminidase inhibition in the ELLA and MUNANA assays. To test this hypothesis, we selected pigs and ferrets with (n =3) and without (n = 3) inhibition of neuraminidase activity in the MUNANA assay to evaluate epitope recognition (all were positive for antibody-mediated neuraminidase inhibition in the ELLA assay) (**Figure 6A**). We normalized total anti-NA antibody input between animals based on anti-NA IgG ELISA titers. This normalization step allows for a better comparison of specific epitope targeting that is not confounded by overall magnitude of humoral responses to the DNA vaccine, which varied between animals. Due to reagent availability, serum antibody binding for individual animals was only assessed on the cyclic constrained microarray.

Consistent with pooled serum, the individual serum antibodies targeted primarily the catalytic head in both pigs and ferrets (**Figure 6B**). The different animals displayed highly individual binding profiles with limited overlap in the targeted peptides (**Figure 6C**). Yet one unique epitope, DTVGWSWPDGAEL, was recognized both post-vaccination and post-challenge by all three animals with neuraminidase inhibition in the MUNANA assay. Ferret 31 serum antibodies, which did not display MUNANA inhibition at any time point, recognized this epitope post-vaccination, but not post-challenge. Serum antibodies from Pig 4 and Ferret 31 (with no MUNANA inhibition), did not bind the DTVGWSWPDGAEL peptide, but rather a peptide that partly overlaps the aforementioned peptide; SWPDGAELPFTID. The location of the DTVGWSWPDGAEL epitope is predicted to the catalytic head of NA, at the interface of the monomers in the tetramer, and is not part of the active site, second sialic binding site, nor contain framework residues (**Figure 6D**). The three animals that did not display NA inhibition in the MUNANA assay (Ferret 31, Ferret 35 and Pig 4) all bound the RPWVSFNQNLEYQ peptide. In contrast, serum antibodies from none of the animals with MUNANA inhibition (Pig 3, Pig 5, and Ferret 23) bound this peptide.

## 4 Discussion

Vaccine-induced antibodies should ideally target critical functions of the virus to protect against infection. While antibodies directed against NA do not inhibit virus entry into the host cell (non-neutralizing), they play an important role in influenza disease and are associated with less severe disease outcomes and reduced virus shedding and symptoms (14, 15, 33–35). Here, we define the functionality of DNA vaccine-induced NA-specific antibodies and associated B cell epitopes on the NA of the A/California/04/2009(H1N1)pdm09 influenza virus in DNA vaccinated and challenged pigs and ferrets. All animals developed NA-specific IgG antibodies post-vaccination that persisted post-challenge. However, the ability of serum anti-NA antibodies to inhibit neuraminidase enzymatic activity differed notably between pigs and ferrets, depending on the assay used. In particular, serum antibodies from both animal species could inhibit neuraminidase cleavage of the large sialylated glycoprotein used in the ELLA assay, whereas only pig serum antibodies could inhibit neuraminidase cleavage of the small substrate used in the MUNANA assay. Epitope mapping of serum antibodies on linear and cyclic constrained peptide microarrays highlight marked differences in epitope recognition by pig and ferret sera following DNA vaccination and viral challenge, which may account for the species-specific differences observed for antibody-mediated neuraminidase inhibition in the MUNANA assay.

The MUNANA method is commonly used for screening of NA drug-resistance and is based on the cleavage of a small synthetic substrate (36). In contrast, ELLA uses fetuin as substrate, a natural, bulky glycoprotein containing abundant neuraminic acids, including α2,3-linked sialic acid and α2,6-linked sialic acid (36). Considering the differences in substrates, it is plausible that antibodies directed towards the active site may be necessary to inhibit cleavage of the MUNANA substrate. Conversely in the ELLA assay, antibodies inhibiting neuraminidase cleavage of the bulkier glycans on fetuin may do so mechanistically through stearic hindrance in addition to direct binding to critical sites, such as the active site and second sialic acid binding site (19). HA-specific antibodies can also sterically interfere with NA activity (31, 32). To minimize the potential interference of HA-specific antibodies in the MUNANA and ELLA assays, we used a reassortant virus strain with an HA distant to the HA of A/California/04/2009(H1N1)pdm09 in the DNA vaccine. Since fetuin, with its neuraminic acids, may be more representative of sialylated glycoproteins in the influenza virus host, the ELLA assay may arguably be more appropriate for measuring antibody-mediated neuraminidase inhibition.

Through epitope mapping using peptide microarrays, we identified distinct B cell epitopes in pig and ferret sera, which may contribute to the marked differences observed for antibody-mediated inhibition in two functional NA assays. The limited targeting of known critical functional or structural residues on the NA protein by ferret serum antibodies may explain, in part, the inability of the ferret sera to inhibit NA cleavage of the small substrate used in the MUNANA assay. However, despite only six regions targeted by antibodies on the linear and cyclic constrained microarrays combined, ferret sera post-challenge demonstrated an increased ability to inhibit neuraminidase activity (ELLA) compared to post-vaccination. Thus, the few regions on the NA protein targeted by ferret antibodies may be sufficient to inhibit neuraminidase cleavage of larger sialylated proteins.

Other factors may potentially contribute to the observed differences in B cell epitope recognition between the DNA vaccinated pigs and ferrets. Firstly, both animal species received the same influenza DNA vaccine by means of the IDAL needle-free jet-injection device. This administration device forces a hair-thin stream of liquid into the dermis, which is rich in antigen presenting immune cells, through carefully calibrated pressure specific for pig skin. However, there are marked differences between pig skin and ferret fur skin that include thickness, stiffness, extent of underlying fat, and hair density. It is therefore possible that, when administered with the IDAL device optimized for pig skin, the DNA vaccine enters different anatomical compartments in pigs and ferrets, which may affect the vaccine-induced humoral immune response. Secondly, the vaccination regimens and relative timing of serum sampling was different between the pig and ferret studies. The pigs received two vaccinations compared to three for the ferrets and both the interval between vaccinations and subsequent serum sampling was one week longer in the pig study. Thirdly, the pigs received a higher dose of vaccine compared to the ferrets (800 µg and 500 µg vs. 300 µg), based on the different size of the two species. The different doses in the pigs showed a clear dose-dependent antibody response. Collectively, or independently, these factors may have lowered the influenza DNA vaccine-induced antibody response below our stringent cut-off level for positive peptide recognition.

By identifying common epitopes targeted in individual animals with differential NA inhibition in the ELLA and MUNANA assays, we identified a common epitope, DTVGWSWPDGAEL, associated with antibody-mediated neuraminidase inhibition in the MUNANA assay. Its predicted location is in the NA monomer interface and may be solvent accessible. Intriguingly, the epitope does not contain residues of the active site or second sialic binding site, although one residue, Trp-456, has been identified as one out of twelve critical antigenic residues of the N1 protein that confers protection against lethal influenza challenge in mice (37). However, it is uncertain if this single critical residue can solely explain the observed NA inhibition. The antibodies targeting this epitope sequence inhibit NA activity through steric hindrance or by affecting necessary mobility of the protein during binding and cleavage. For example, a monoclonal antibody (CD6) targeting a large epitope spanning two adjacent NA monomers of the A/California/04/2009(H1N1)pdm09 influenza that does not comprise residues of the active site or the second sialic binding site could inhibit the enzymatic activity of NA in ELLA through steric hindrance (38). Similarly, the DTVGWSWPDGAEL epitope identified in the present study does not contain critical residues of the active site, second sialic binding site or framework, but it was identified in both ferrets and pigs with NA inhibition in both inhibition assays. Furthermore, this epitope, DTVGWSWPDGAEL, identified on NA of A/California/04/2009(H1N1)pdm09 share complete sequence homology with NA of A/Vietnam/PEV16T/2005, which is the closest available sequence to the H5N1 challenge strain. (**Supplementary Figure 1**). Targeting of this epitope by vaccine-induced antibodies may have contributed to the vaccine-induced protection against disease severity observed in ferrets after lethal challenge with avian H5N1. It is therefore possible that the DTVGWSWPDGAEL epitope may be an important antigenic site and possibly play a role in blocking the enzymatic activity of NA. The extent to which human NA-specific antibodies target this epitope is unknown. Since seasonal influenza vaccines are poor inducers of anti-NA antibodies compared to natural infection, we anticipate that antibodies specific for DTVGWSWPDGAEL may be absent in individuals vaccinated with current seasonal vaccines, but potentially present in those who had a natural infection (39). The potential functional significance of this epitope warrants further investigation in humans.

One of the epitopes, NNILRTQESECAC, recognized in DNA vaccinated pigs, contains a sequence that is conserved for NAs in almost all influenza A subtypes and influenza B (40, 41). This epitope is located between residues 222-230 (N2 numbering) and includes active site and framework residues (Ile-222, Arg-224 and Glu-227) (42). Antibodies raised against this epitope can inhibit the activity of almost all NAs of influenza A subtypes (N1-9) as well as NAs from influenza B (41, 42). In our study, antibody binding to this epitope was boosted in vaccinated pigs after infection and showed IgG recognition for adjacent amino acid sequences as well. This epitope sequence was also identified in two individual pigs post-vaccination. The significance of this epitope to NA function makes it an attractive target in rational vaccine design, and anti-NA antibodies directed against this sequence may have the potential to confer broad protection against plentiful influenza A and B subtypes.

DNA vaccine-induced antibodies also targeted epitopes with residues within the enzymatic active site, the second sialic binding site and framework. In particular, the pig post-vaccination and post-challenge serum antibodies targeted two cyclic constrained peptides NDKHSNGTIKDRS and DRSPYRTLMSCPI containing the Asp-151 and Arg-152 active site residues and framework residue Arg-156. A linear epitope with the same sequence, NDKHSNGTIKDRSPYRTLMS, was also identified in ferrets post-vaccination and post-challenge. A study of B cell epitopes in Pandemrix^®^ vaccinated and unvaccinated humans during the 2009 influenza season identified an analogous but slightly shorter epitope, NDKHSNGTIKDRSPY (43). IgG recognition of this epitope was stronger in unvaccinated individuals after influenza infection compared to vaccinated individuals (43). This region may thus be a potent antigenic site of NA in various animal species and the functional role of anti-NA antibodies binding to this epitope warrants further investigation.

## 5 Conclusion

We evaluated the functional responses of NA-specific antibodies and defined antigenic sites on N1 of pandemic influenza virus A/California/04/2009(H1N1)pdm09 in pigs and ferrets vaccinated with our influenza DNA vaccine. The vaccine-induced antibodies were able to block the catalytic activity of NA. We saw a differential recognition pattern in the two animal species, though with the presence of few common epitopes. We defined a specific antigenic site only seen in individual animals with NA inhibition in two inhibition assays. Unexpectedly, this antigenic site did not contain residues of the catalytic or structural site of NA, but could possibly block the activity through steric hindrance. Further studies investigating the potential role of this epitope in NA inhibition is warranted, but is beyond the scope of this study. Overall, our findings showed that the NA-specific antibodies induced by the vaccine target critical antigenic sites essential for the catalytic and structural function of NA.

## Data availability statement

The original contributions presented in the study are included in the article/**Supplementary Material**. Further inquiries can be directed to the corresponding author.

## Ethics statement

Pig Study: The ethics Committee for Animal Experimentation from the Institute of Agrifood Research and Technology and the Animal Experimentation Commission from the Autonomous Community of Catalonia Government in compliance with the Directive, UE 63/2010 and the Spanish Legislation, RD53/2013 and the Catalan Law 5/1995 and Decree 214/1997. Ferret study: All husbandry and procedural work was conducted by NIBSC animal technicians in accordance with UK Home Office license numbers 80/2537 and PCB39ED3C, under Protocol numbers 4 and 5.

## Author contributions

JT, RL, AF, and CP designed the study. OB, CR, and CS designed and produced the linear peptide microarray. JT, RL, CR, and CS performed experiments. JT, RL, and CP performed data and statistical analysis. JT, CP and RL drafted the manuscript. RL, CP, AF, and IJ supervised the study. All authors contributed to the article and approved the submitted version.
