# Supplementary material for: Differential recognition of influenza A virus H1N1 neuraminidase by DNA vaccine-induced antibodies in pigs and ferrets
Source: Front Immunol. 2023 May 29;14:1200718. doi: 10.3389/fimmu.2023.1200718 (PMC10258320; doi:10.3389/fimmu.2023.1200718)
Supplement: Supplementary file 1 [file Image_1.pdf]

```
H5N1      MNPNQKIITIGSICMVTGIVSLMLQVGNMISIWVSHSIHTGSQHQAEP------------      48
H1N1      MNPNQKIITIGSVCMTIGMANLILQIGNIISIWISHSIQLGNQNQIETCNQSVITYENNT      60
          ************:**. *:..*:**:**:****:****: *.*:* *

H5N1      --------ISNTNFLTEKAVASVKLAGNSSLCPINGWAVYSKDNSIRIGSKGDVFVIREP      100
H1N1      WVNQTYVNISNTNFAAGQSVVSVKLAGNSSLCPVSGWAIYSKDNSVRIGSKGDVFVIREP      120
                ****** : ::*.************:.***:******:**************

H5N1      FISCSHLECRTFFLTQGALLNDKHSNGTVKDRSPHRTLMSCPVGEAPSPYNSRFESVAWS      160
H1N1      FISCSPLECRTFFLTQGALLNDKHSNGTIKDRSPYRTLMSCPIGEVPSPYNSRFESVAWS      180
          ***** **********************:*****:*******:**.**************

H5N1      ASACHDGTSWLTIGISGPDNGAVAVLKYNGIITDTIKSWRNNILRTQESECACVNGSCFT      220
H1N1      ASACHDGINWLTIGISGPDNGAVAVLKYNGIITDTIKSWRNNILRTQESECACVNGSCFT      240
          ******* .***************************************************

H5N1      VMTDGPSNGQASHKIFKMEKGKVVKSVELDAPNYHYEECSCYPDAGEITCVCRDNWHGSN      280
H1N1      VMTDGPSNGQASYKIFRIEKGKIVKSVEMNAPNYHYEECSCYPDSSEITCVCRDNWHGSN      300
          ************:***::****:*****::**************:.**************

H5N1      RPWVSFNQNLEYQIGYICSGVFGDNPRPNDGTGSCGPVSSNGAYGVKGFSFKYGNGVWIG      340
H1N1      RPWVSFNQNLEYQIGYICSGIFGDNPRPNDKTGSCGPVSSNGANGVKGFSFKYGNGVWIG      360
          ********************:********* *********** *****************

H5N1      RTKSTNSRSGFEMIWDPNGWTETDSSFSVKQDIVAITDWSGYSGSFVQHPELTGLDCIRP      400
H1N1      RTKSISSRNGFEMIWDPNGWTGTDNNFSIKQDIVGINEWSGYSGSFVQHPELTGLDCIRP      420
          **** .**.************ **..**:*****.*.:**********************

H5N1      CFWVELIRGRPKESTIWTSGSSISFCGVNSDTVGWSWPDGAELPFTIDK 449
H1N1      CFWVELIRGRPKENTIWTSGSSISFCGVNSDTVGWSWPDGAELPFTIDK 469
          *************.***********************************
```

Supplementary figure 1: Sequence alignment of NA protein from A/California/04/09(H1N1)pdm09 and A/Vietnam/PEV16T/2005(H5N1). Amino acid sequence alignment between NA from avian A/Vietnam/PEV16T/2005(H5N1) and the vaccine strain A/California/04/09(H1N1)pdm09. The epitope targeted by vaccine-induced antibodies post-vaccination and post-challenge in individual animals with NI activity, DTVGWSWPDGAEL, is shown in bold and blue shading.
